# Treatment Patterns, Clinical Outcomes and Quality of Life in BRCA1/2-Associated Breast Cancer Patients: A Retrospective Analysis

**DOI:** 10.3390/curroncol32050269

**Published:** 2025-05-02

**Authors:** Anna-Maria Parger, Paulina Gebhart, Daniela Muhr, Christian F. Singer, Yen Y. Tan

**Affiliations:** Department of Obstetrics and Gynaecology, Comprehensive Cancer Center, Medical University of Vienna, Waehringer Guertel 18-20, 1090 Vienna, Austria; n0842400@students.meduniwien.ac.at (A.-M.P.); paulina.gebhart@meduniwien.ac.at (P.G.); daniela.muhr@meduniwien.ac.at (D.M.); christian.singer@meduniwien.ac.at (C.F.S.)

**Keywords:** BRCA mutation, breast cancer, treatment regimens, clinical outcomes, retrospective analysis, quality of life

## Abstract

**Background**: Breast cancer (BC) patients with germline BRCA1/2 pathogenic variants (PVs) often face unique challenges compared to non-carriers. However, the impact of PVs on treatment patterns, clinical outcomes, and quality of life (QoL) remains insufficiently explored. This study aims to assess these factors in these individuals. **Methods**: A retrospective analysis was conducted using data from the Medical University of Vienna Center for Familial Breast and Ovarian Cancer between 2011 and 2021. Among 1285 individuals identified, 338 were included (120 BRCA1 PVs, 47 BRCA2 PVs, and 171 non-carriers). Clinical data including treatment patterns and outcomes were collected; QoL was assessed in BRCA1/2 PV carriers using the SF-12 questionnaire. **Results**: Among 338 BC patients, BRCA1 PV carriers were significantly younger at disease onset and more likely to present with triple-negative BC, with higher Ki-67 (>10%) than BRCA2 or non-carriers. Platinum-based chemotherapy was more frequently administered to BRCA PV carriers for neoadjuvant treatment (OR 7.7, *p* < 0.001), and therapeutic bilateral mastectomy was more common in BRCA1 carriers (44.7%) compared to BRCA2 (37.8%, *p* = 0.114) and non-carriers (25.2%, *p* = 0.003). Epirubicin was the primary agent for adjuvant chemotherapy across all groups compared to other chemotherapeutic agents. QoL assessments revealed significant physical health challenges, particularly among those who underwent neoadjuvant chemotherapy and surgery, while mental health scores remained relatively high. **Conclusions**: This study highlights the distinct treatment patterns and tumor characteristics associated with BRCA1/2 carriers, including the impact of treatments on quality of life. Nevertheless, our findings ought to be interpreted with caution due to the small sample size. Larger prospective studies with more complete treatment data, including PARP inhibitor use, and further research on supportive care strategies are needed for this high-risk population.

## 1. Introduction

Breast cancer (BC) is the most common cancer affecting women, with up to 10% of cases attributed to hereditary gene predispositions, such as *BRCA1* and *BRCA2* mutations. Women who inherit these mutations have a high lifetime risk for BC and ovarian cancer (OC) [1]. While BRCA2-associated cancers are predominantly hormone receptor-positive tumors, BRCA1-associated tumors are more frequently classified as triple-negative breast cancer (TNBC), a subtype known for its aggressive nature and higher sensitivity to neoadjuvant chemotherapy [2,3]. Targeted treatments like PARP inhibitors have demonstrated efficacy in BRCA mutation carriers by exploiting defects in DNA repair pathways, leading to improved clinical outcomes. Prophylactic surgeries, such as bilateral mastectomy and salpingoophorectomy, are routinely offered to these patients to mitigate the risks of cancer onset and recurrence.

Despite advances in treatment, the clinical and psychological outcomes for BRCA1/2 carriers remain uncertain. Previous studies have reported better, worse or similar outcomes for patients with BRCA1 or BRCA2 germline pathogenic variant (PV) compared to non-carriers [4,5,6,7,8,9,10]. Some evidence suggests that BRCA1/2 PV carriers may initially respond better to DNA-damaging agents, such as platinum chemotherapy [3,11], although long-term survival benefits are still being debated [12]. Importantly, patient quality of life (QoL), a crucial aspect of cancer survivorship, has shown divergent trends. For example, some studies report better QoL with treatments like PARP inhibitors instead of chemotherapy [13,14,15], while others highlight significant physical and mental health challenges following treatment and prophylactic surgeries [14,16,17,18].

Although the biological differences between BRCA1- and BRCA2-associated breast cancers have been extensively studied, there is a lack of studies investigating how different treatment regimens influence QoL outcomes in this population. It is also unclear whether different treatment strategies, such as neoadjuvant versus adjuvant chemotherapy, influence the physical and mental health of these patients in a meaningful way. Addressing this gap, our study aims to analyze treatment patterns and clinical outcomes among BRCA1/2 pathogenic variant (PV) carriers and patients without PV (i.e., wild-type, WT), and to compare QoL outcomes between BRCA1 and BRCA2 carriers. This study seeks to provide insights into the impact of genetic status on treatment-related outcomes and survivorship experiences.

## 2. Material and Methods

### 2.1. Study Design and Participants

We conducted a retrospective analysis of high-risk breast cancer patients who underwent genetic testing at the Medical University of Vienna Familial Breast and Ovarian Cancer Centre between 2011 and 2021. Patients were identified through the clinic’s prospective hereditary cancer registry, ATHENA, and were eligible for inclusion if they were 18 years or older, attended genetic counselling and testing, consented to research participation, had a confirmed BRCA1/2 variant, and had available treatment data. The primary reason for exclusion was missing treatment data. Ethical approval for the study was obtained from the Medical University of Vienna Ethics Committee (EK2190/2019, EK1869/2020). All methods were performed in accordance with standard guidelines and regulations.

### 2.2. Data Collection

Clinical and treatment data, including histopathological characteristics, chemotherapy regimens and surgical procedures, were collected manually from patient records or electronically from the hospital database between November 2020 and February 2023. Survival outcomes, such as dead or alive, were obtained from Statistics Austria.

For clarity, surgical procedures performed after a breast cancer diagnosis for treatment purposes are referred to as therapeutic mastectomies, while those performed to reduce cancer risk in high-risk individuals are referred to as prophylactic mastectomies throughout this manuscript. PARP inhibitors were available only via clinical trials during the study period (2011–2021).

For QoL assessment, only BRCA1/2 PV carriers were invited to complete the Short Form Survey (SF-12) questionnaire by post. The SF-12 is a validated instrument for measuring health-related quality of life (HRQoL) across two key domains: physical and mental health [19]. It consists of 12 items, which are divided into two summary scores, i.e., Physical Component Summary (PCS-12) and the Mental Component Summary (MCS-12). Each summary score ranges from 0 to 100, with higher scores representing better perceived health status and overall quality of life [19,20]. Lower scores reflect greater impairment. Specifically, a PCS-12 score of 50 or less suggests physical health limitations, while an MCS-12 score of 42 or less suggests potential mental difficulties [20,21]. These thresholds help identify patients who may benefit from additional medical or psychosocial support.

The invitation package was sent in April 2021 and included a cover letter, patient consent form and the questionnaire. Non-responders were followed up twice after initial mailing, i.e., at two weeks and four weeks.

### 2.3. Statistical Analyses

Descriptive statistics were used to summarize demographic, clinical and treatment data. Categorical variables were expressed as absolute frequencies and percentages. Continuous variables were summarized using mean, median, standard deviation, minimum and maximum. The median follow-up time was calculated from the date of breast cancer diagnosis to the last documented follow-up or death. Time to local recurrence or contralateral breast cancer diagnosis were calculated from the date of breast cancer diagnosis to the date of documented recurrence or contralateral breast cancer diagnosis.

BRCA mutation status is divided into 3 categories: BRCA1, BRCA2 and BRCA wild-type (WT). Differences between groups were analyzed using the following methods: Continuous variables: analysis of variance (ANOVA) or Kruskal–Wallis test for non-normal distributions, followed by Bonferroni corrected post hoc analyses. Categorical variables: Chi-squared test or Fisher’s exact test or smaller sample sizes; any differences were followed up with Bonferroni correction.

Statistical significance was defined as *p* < 0.05; all analyses were performed using IBM SPSS Statistics version 29. Results were graphically presented using boxplots and bar charts.

## 3. Results

### 3.1. Cohort Description

Table 1 shows the cohort description. Of 1285 women identified in the registry, 338 met the inclusion criteria and were included in the study. A total of 120 were BRCA1 PV carriers (35.5%), 47 were BRCA2 PV carriers (13.9%) and 171 were non-carriers (wild-type, WT) (50.6%). The median follow-up time for the cohort was 7 years (IQR: 2–10 years).

The median age of breast cancer onset differs significantly between BRCA1 carriers (41 years; range 22–74) compared to WT carriers (48 years; range 27–78; *p* < 0.001). No significant differences were observed between BRCA2 carriers and other groups in terms of age at onset. BRCA1 PV carriers had a significantly higher risk of developing second breast or ovarian cancer than BRCA2 or WT carriers (*p* = 0.010).

### 3.2. Histopathological Characteristics

**Figure 1:**
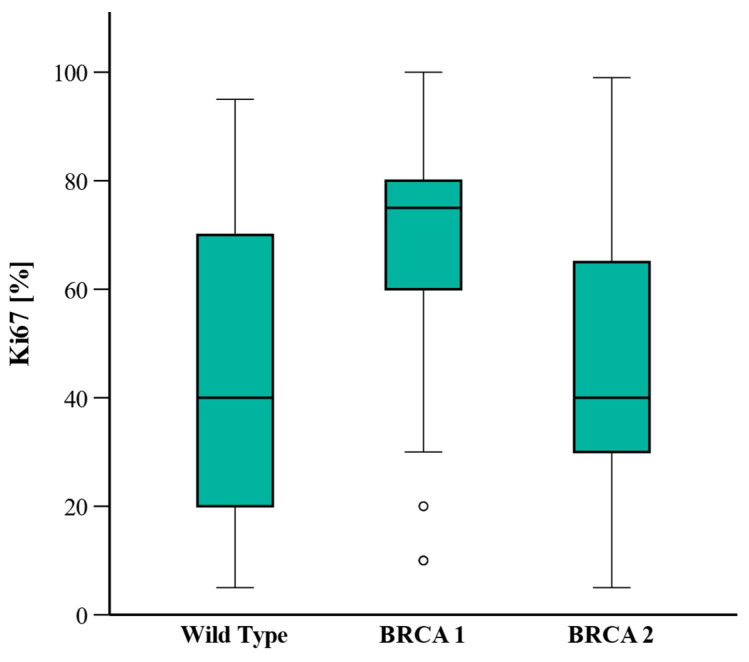
Ki-67 level by *BRCA* mutation status.

BRCA1 PV carriers were significantly more likely to be diagnosed with TNBC and higher Ki-67 (>10%) (*p* < 0.001), indicating a more aggressive tumor type. In contrast, BRCA2 PV and WT carriers were mostly diagnosed with hormone receptor-positive/HER2-negative tumors with lower Ki-67 values (Figure 1).

### 3.3. Treatment Regimens

**Figure 2:**
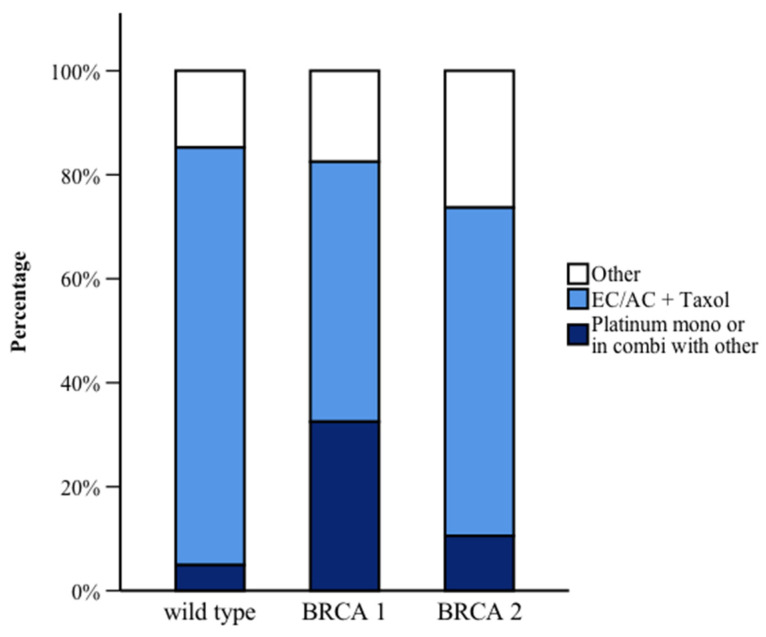
Neoadjuvant treatments by *BRCA* mutation status. Abbreviations: EC (Epirupicine; Cyclophosphamide); AC (Adriamycine; Cyclophosphamide); Taxol (Paclitaxel); Platinum (Cisplatin or Carboplatin).

Table 2 shows the different treatment regimens by mutation type. For neoadjuvant chemotherapy, BRCA1/2 PV carriers were significantly more likely to receive platinum-based chemotherapy compared to WT carriers (odds ratio (OR) 7.7, 95% confidence interval (CI) 2.1–28.6; *p* < 0.001) (Figure 2). However, there were no significant differences between the groups regarding the total number of chemotherapy cycles or pathologic complete response (pCR) rates.

Adjuvant chemotherapy was more common in BRCA1 carriers than in WT carriers (40.9% vs. 18.7%; *p* < 0.001). Anti-hormonal treatments were more frequently prescribed to BRCA2 and WT carriers, aligning with their hormone receptor-positive tumor subtypes. Epirubicin was the primary agent for adjuvant chemotherapy across all groups (39% WT, 32% BRCA1 and 64% BRCA2 carriers) compared to other chemotherapeutic agents, *p* = 0.247).

### 3.4. Surgical Interventions

**Figure 3:**
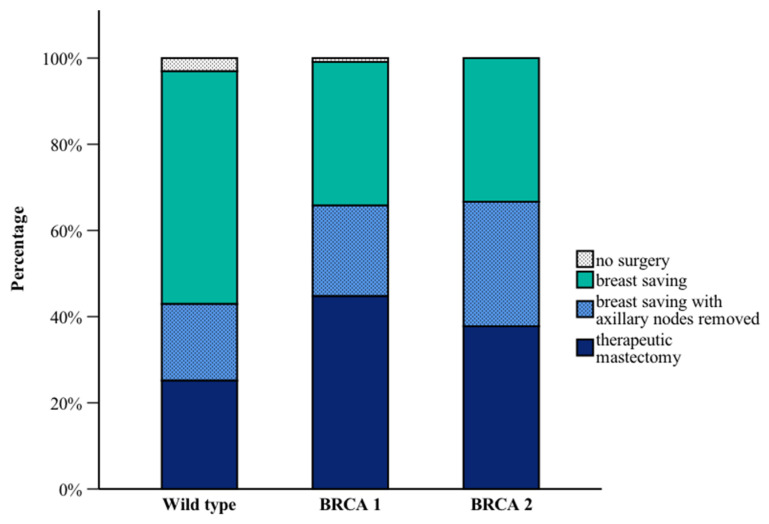
Type of surgeries by *BRCA* mutation status.

BRCA1 PV carriers underwent therapeutic mastectomy more often than BRCA2 PV carriers (44.7% vs. 37.8%; *p* = 0.114) and WT carriers (44.7% vs. 25.2%; *p* = 0.003) (Figure 3). No significant differences were observed regarding recurrence, tumor size or lymph node involvement across the groups.

### 3.5. Quality of Life

**Figure 4:**
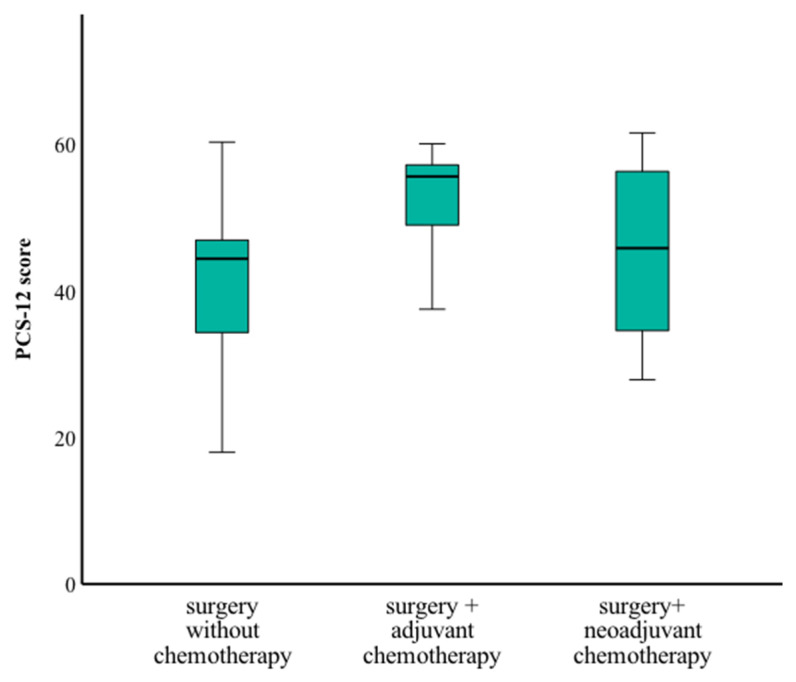
Physical health score by treatment type.

Fifty BRCA PV carriers completed the SF-12 questionnaire, with an average response time of six years after BC diagnosis. Physical health scores were lower among BRCA1 and BRCA2 carriers, indicating physical limitations, but no significant differences were observed between groups (*p* = 0.201) (Figure 4). Mental health scores were comparable across groups and indicated relatively good mental well-being (*p* = 0.773).

**Figure 5:**
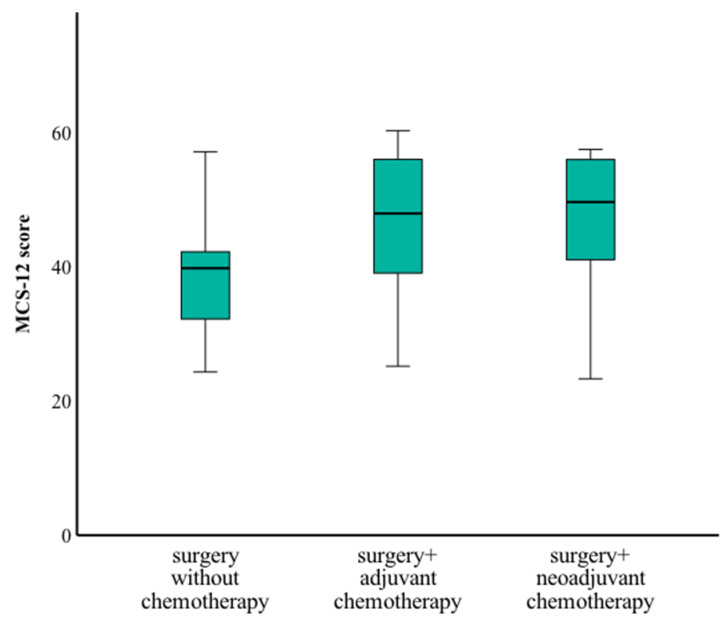
Mental health score by treatment type.

Patients who underwent surgery followed by adjuvant chemotherapy reported significantly better physical scores than those treated with neoadjuvant chemotherapy prior to surgery or surgery alone (*p* = 0.024) (Figure 5). There were no significant differences in mental health scores based on treatment regimen.

## 4. Discussion

In this study, we analyzed a comprehensive set of data on diagnosis, treatment and quality of life (QoL) of high-risk breast cancer patients. Our findings show distinct tumor biology, treatment patterns and survivorship challenges in BRCA PV carriers.

BRCA1 PV carriers were significantly younger at cancer onset and presented with more aggressive tumor subtypes, such as TNBC and higher Ki-67 values, than BRCA2 PV or WT carriers. These results are consistent with other studies, reaffirming that BRCA1 PV carriers are more likely to develop cancer earlier in life and with biologically more aggressive tumor subtypes [3,22,23,24,25]. Interestingly, BRCA1 PV carriers also exhibited a higher risk of developing contralateral breast cancer and ovarian cancer after an initial breast cancer diagnosis. This result is also consistent with a prior study where the authors reported a 2.7-fold increased risk of CBC in BRCA1 PV carriers compared to non-carriers, with a hazard ratio of 2.7 (95% CI, 2.0–3.7) [26]. These findings emphasize the importance of early genetic testing and regular surveillance in high-risk populations to enable timely intervention.

Our analysis of treatment patterns revealed that BRCA1/2 PV carriers were significantly more likely to receive platinum-based neoadjuvant chemotherapy. This aligns with existing evidence supporting the efficacy of platinum agents in BRCA-associated tumors due to their targeting of homologous recombination repair defects [27]. Recent studies, such as the TNT trial, demonstrated that BRCA1/2 mutation carriers derive greater benefit from platinum-based chemotherapy compared to taxane-based regimens, with higher response rates and improved progression-free survival [11]. However, no significant differences were observed in pCR rates, tumor size or lymph node involvement between BRCA1-, BRCA2-PV and WT carriers, suggesting that the choice of treatment regimen does not necessarily translate into superior clinical outcomes. This highlights the complexity of treatment responses, where genetic and biological differences may not always translate to clinical advantages in terms of complete remission.

Management practices observed in our cohort largely align with earlier guideline-based care but predate the broader incorporation of newer standards, including adjuvant PARP inhibitor therapy. Current guidelines, such as those from the NCCN and ESMO, now recommend the use of adjuvant PARP inhibitors for high-risk BRCA1/2 mutation carriers following evidence from the OlympiA trial [6,7]. Furthermore, recent consensus statements highlight the importance of integrating genetic testing results into clinical decision-making to tailor systemic therapy and surgical strategies [25].

Notably, this study highlights a gap in the use of PARP inhibitors, which have shown promise in improving survival and QoL in BRCA1/2 carriers. Since the OlympiA trial establishing the role of adjuvant PARP inhibitors only matured in 2021 [28], they were not part of standard clinical care during most of our study period (2011–2021). Therefore, any use in our cohort likely occurred within clinical trials. The limited use reflects both the timing of emerging evidence and the retrospective design of this study. The OlympiA trial also demonstrated that adjuvant olaparib significantly improves invasive disease-free and overall survival in patients with germline BRCA1/2 mutations and high-risk HER2-negative early breast cancer [28,29]. These findings have led to the integration of PARP inhibitors into treatment guidelines for BRCA-associated breast cancer, underscoring the importance of genetic testing in guiding therapy choices.

In terms of surgical approaches, BRCA1 PV carriers were more likely to undergo therapeutic mastectomies compared to BRCA2 PV or WT carriers, a finding corroborated by previous studies [30,31]. The higher mastectomy rates in BRCA1 PV carriers may reflect both the aggressive nature of their tumors and the proactive surgical decision to reduce future cancer risk. Prophylactic mastectomy is usually recommended to BRCA1/2 PV carriers so that patients could opt for such intervention as a preventive strategy [32].

A unique aspect of this study is its focus on QoL outcomes. Despite the small sample size, BRCA1/2 PV carriers reported significant physical health challenges, as reflected in lower physical scores, particularly among those who underwent neoadjuvant chemotherapy prior to surgery or surgery alone. Interestingly, patients who received adjuvant chemotherapy reported better physical health scores compared to those who underwent neoadjuvant chemotherapy. This difference may reflect the more aggressive and prolonged treatment courses often associated with neoadjuvant regimens, as well as more aggressive diseases at presentation. In contrast, patients treated with adjuvant therapy may have had a smoother recovery following surgery, contributing to better physical health outcomes. These findings emphasize the need to consider survivorship care plans that address the physical burdens of treatment, including rehabilitation and supportive care strategies.

Several recent studies have shown that although platinum-based chemotherapy and PARP inhibitors improve oncologic outcomes, they are associated with increased rates of fatigue, hematologic toxicities and gastrointestinal symptoms, which can negatively impact physical health [13,14,16,33,34]. Nonetheless, mental health often remains stable or improves over time, particularly with structured survivorship support [18]. By contrast, the mental scores in our cohort were relatively high, suggesting resilience or adequate psychological support. However, this finding must be interpreted with caution, as the questionnaires were administered a considerable amount of time after surgery, potentially dampening the immediate psychological effects of surgery due to the passage of time.

Future research should focus on longitudinal QoL assessments to address the impact of lag-time bias to better understand and improve the physical and mental well-being of BRCA PV carriers. The findings also call for a more tailored approach to treatment and survivorship care. For example, incorporating an exercise program, personalized physiotherapy, psycho-oncology support, nutritional counselling, mindfulness training and peer support groups through a multidisciplinary care pathway could promote holistic recovery and well-being of BRCA PV carriers. At our institute, survivorship support is integrated into clinical care, including genetic counselling, psycho-oncology services and follow-up care pathways for BRCA1/2 carriers and their family members. Similar multidisciplinary survivorship models have been increasingly adopted in other European centers and are increasingly recognized as critical components of comprehensive cancer care [35,36].

Despite these insights, the study has limitations. As a retrospective analysis, it relied on existing clinical records, many of which were incomplete. As a result, a substantial number of patients were excluded due to missing data, leading to smaller subgroup sizes and reduced statistical power to detect meaningful differences. Future prospective studies with more complete datasets are needed to validate these findings and to investigate how emerging treatments, such as PARP inhibitors and immunotherapies, impact clinical outcomes and QoL in high-risk population.

## 5. Conclusions

This study provides valuable insights into the relationship between treatment patterns, clinical outcomes and quality of life of BRCA-associated breast cancer patients. While our results show higher use of platinum-based chemotherapy and therapeutic bilateral mastectomy in BRCA1 PV carriers, the remission and survival rates were comparable across groups. QoL assessments revealed lower physical health scores in BRCA carriers undergoing aggressive treatments, with mental health scores remaining relatively high, suggesting preserved psychological well-being despite physical health challenges. These findings should be interpreted with caution given the limited sample size, particularly for the QoL analysis. Larger prospective studies with more complete treatment data, including PARP inhibitor use, are needed to validate these results and further inform tailored treatment and supportive care strategies for this population.

## Figures and Tables

**Table 1:** Cohort description.

		BRCA-WT N (%) 171 (50.6%)	BRCA1 N (%) 120 (35.5%)	BRCA2 N (%) 47 (13.9%)	*p*-Value
Age at disease onset, median years		48.0	41.5	46.0	Post hoc: WT vs. BRCA1: <0.001 WT vs. BRCA2: 0.178 BRCA1 vs. 2: 0.080
Age at local recurrence, median years		58.0	50.0	54.5	0.071
Age at contralateral BC, median years		61.0	47.0	52.5	Post hoc: WT vs. BRCA1: 0.026 WT vs. BRCA2: 0.999 BRCA1 vs. 2: 0.993
Molecular subtype	TNBC	56 (34.6)	75 (78.1)	12 (31.6)	Post hoc: WT vs. BRCA1: <0.001 WT vs. BRCA2: 0.999 BRCA1 vs. 2: <0.001
	ER/PR-pos, HER2 neg	72 (44.5)	16 (16.7)	20 (52.6)	
	HER2-pos	25 (15.4)	5 (5.2)	4 (10.5)	
	ER-pos/PR-neg/HER2-neg	9 (5.5)	0	2 (5.3)	
Ki-67, percent (range)		40 (20–70)	75 (60–80)	40 (30–70)	Post hoc: WT vs. BRCA1: <0.001 WT vs. BRCA2: 0.561 BRCA1 vs. 2: 0.591
Time to local recurrence, median months (range)		130.0 (12–407)	93.5 (16–258)	97.0 (25–288)	0.661
Time to contralateral BC, median months (range)		132.0 (22–373)	106.0 (25–186)	124.5 (25–172)	0.502
pCR	Complete response	16 (29.6)	19 (45.2)	7 (41.2)	
	Partial response	28 (51.9)	22 (52.3)	6 (35.3)	0.070
	No response	10 (18.5)	1 (2.5)	4 (23.5)	
Deceased		22 (12.9)	16 (13.3)	6 (12.8)	0.992

**Abbreviations:** WT: wild-type; BRCA1: breast cancer gene 1; BRCA2: breast cancer gene 2; BC: breast cancer; TNBC: triple negative breast cancer; ER: estrogen receptor; PR: progesterone receptor; HER2 neg: human epidermal growth factor receptor 2 negative; HER2 pos: human epidermal growth factor receptor 2 positive; Ki-67: nuclear protein marker of cell proliferation; pCR: pathological complete response.

**Table 2:** Treatment regimens by *BRCA* mutation status.

	BRCA-WT N (%) 171 (50.6%)		BRCA1 N (%) 120 (35.5%)	BRCA2 N (%) 47 (13.9%)	*p*-Value
Adjuvant chemotherapy	no	135 (81.3)	65 (59.1)	29 (69.0)	Post hoc WT vs. BRCA1: <0.001 WT vs. BRCA2: 0.246 BRCA1 vs. BRCA2: 0.756
	yes	31 (18.7)	45 (40.9)	13 (31.0)	
Neoadjuvant chemotherapy	no	98 (59.4)	55 (51.9)	20 (47.6)	0.270
	yes	67 (40.6)	51 (48.1)	22 (52.4)	
PARP inhibitor ^1^	no	-	105 (95.5)	33 (82.5)	BRCA1 vs. BRCA2: 0.016
	yes	-	5 (4.5)	7 (17.5)	
Anti-hormonal treatment	no	70 (45.2)	88 (83.0)	16 (43.2)	Post hoc WT vs. BRCA1: <0.001 WT vs. BRCA2: 1.000 BRCA1 vs. BRCA2: <0.001
	yes	85 (54.8)	18 (17.0)	21 (56.8)	
Trastuzumab	no	140 (87.5)	109 (98.2)	37 (94.9)	Post hoc: WT vs. BRCA1: 0.006 WT vs. BRCA2: 0.777 BRCA1 vs. BRCA2: 0.831
	yes	20 (12.5)	2 (1.8)	2 (5.1)	
Number of adjuvant chemotherapy cycles	Median (range)	6 (3–9)	6 (2–9)	8 (3–9)	0.584
Number of neoadjuvant chemotherapy cycles	Median (range)	8 (2–8)	7 (3–9)	8 (3–9)	0.178

**Abbreviations:** WT: wild-type; BRCA1: breast cancer gene 1; BRCA2: breast cancer gene 2; BC: breast cancer; PARP: poly (ADP-ribose) polymerase. ^1^ During the study period (2011–2021), PARP inhibitors were not routinely available outside of clinical trials.

## Data Availability

The data presented in this study are available upon request from the corresponding author.
